# Prevalence of *Sarcocystis* infection in processed meat products by using digestion and impression smear methods in Hamedan, Iran

**DOI:** 10.1007/s00580-017-2478-3

**Published:** 2017-05-09

**Authors:** Zainab Sadeghi Dehkordi, Banafsheh Yalameha, Abbas Ali Sari

**Affiliations:** 1 Department of Parasitology, Faculty of Para-Veterinary Science, Bu-Ali Sina University, Hamedan, Iran; 2 Laboratory Science, Faculty of Para-Veterinary Science, Bu-Ali Sina University, Hamedan, Iran; 3 Department of Food Hygiene and Quality Control, Faculty of Para-Veterinary Science, Bu-Ali Sina University, Hamedan, Iran

**Keywords:** *Sarcocystis*, Processed meat-products, Hamedan

## Abstract

*Sarcocystis* is a common zoonotic parasite which can be transmitted through ingestion of contaminated, undercooked meat and is a major cause of economic loss in many countries. This study aimed to detect *Sarcocystis* parasite in processed meat products in Hamedan, Iran. A total of 20 samples of hamburger, sausage, and cocktail were collected from markets from three factories in Hamedan, Iran. The samples were examined by digestion and impression smear methods for detecting *Sarcocystis* parasite. The results showed that 80% of all tested samples were infected with *Sarcocystis*. The infection rate in hamburger, sausage, and cocktail were 87.5, 83.33, and 66.66%, respectively. The highest infestation rate was observed in hamburger. The present study shows that the rate of *Sarcocystis* contamination in meat products is very high. So, evaluation of raw meat quality in addition to applying hygienic programs at all stages of the production line is inevitable. Also, consumption of undercooked meat products or fast food should be avoided.

## Introduction

The genus *Sarcocystis* includes more than 100 species with worldwide distribution. They are protozoan coccidian parasites belonging to the phylum *Apicomplexa* (Fayer 2004). *Sarcocystis* spp. are common parasites of a broad range of vertebrates, including mammals, birds, and fish. Merogony and cyst formation (asexual stage) take place in the intermediate host while gametogony and sporogony (sexual stages) take place in the definitive host. Human as both an intermediate and definitive host is more considerable in the life cycle of this parasite. Most pathogenic *Sarcocystis* spp. like *S*. *humanis* and *S*. *suihumanis* can cause infection in human as intermediate host (Fukuyo et al. 2002). The pathogenicity of *Sarcocystosis* spp. for human is uncertain. In most cases, they are not harmful or may be the cause of mild and transient gastrointestinal signs (Gabriele et al. 2006). The importance of muscular *Sarcocystosis* in farm animals is a well-documented problem. High prevalence of *Sarcocystis* infection is seen in cattle, pig, and sheep in both developing and industrialized countries (Sahl Poulsen and Rune 2014). *Sarcocystosis* in heavily infected animals causes reduced milk production, spontaneous abortions, and death (Fayer 2004). Infected animals can transmit the infection to other hosts via the fecal–oral transmission and leads to nausea, vomiting, and enteritis (acute, chronic, and severe) in human (Fayer et al. 2015). Also, human infection with *Sarcocystis* can be related to ingestion or water contamination with oocysts excreted by carnivorous definitive hosts or eating raw/undercooked meat containing the encysted parasite (Rosenthal et al. 2012). Consumption of processed meat products and fast foods are popular in all over the world (Mehdizadeh et al. 2014). Gabriele et al. found that the prevalence of *Sarcocystis* spp. in Argentinean beef was 33.4%. In Iran, some studies were performed about *Sarcocystis* infection in meat products, for example in the study of Rahdar and Salehi (2011a, b), the rate of *Sarcocystis* infection in hamburger, hotdog, and sausage were seen 56, 20, and 8%, respectively (Rahdar and Salehi 2011b). Up to now, in Hamedan, there is no study about the identification of *Sarcocystis* in meat products. Thus, the aim of this study was to investigate the *Sarcocystis* infection rate in processed meat products in Hamedan,

## Methods

This study was carried out from October 2014 to March 2015; a total of 20 samples, including 8 hamburgers, 6 sausages, and 6 cocktails were collected from 3 plant (A, B, and C) products marketed in Hamadan, Iran and examined by digestion and impression smear (DOB smear) methods. In impression smear method, 2 g of each sample was impressed on the slide and fixed with methanol (70%). All samples were stained by Giemsa staining method and observed microscopically for detecting *Sarcocystis* bradyzoite (Nourollahi Fard et al. 2009). In another method, each sample was digested by both following procedures:

$$ \mathrm{Four}\ \mathrm{g}\mathrm{rams}\ \mathrm{of}\ \mathrm{each}\ \mathrm{sample}\kern0.5em +\kern0.5em 3.5\;\mathrm{mL}\ \mathrm{HCl}\kern0.5em +\kern0.5em 2.5\;\mathrm{g}\ \mathrm{trypsin}\kern0.5em +\kern0.5em 1500\;\mathrm{mL}\ \mathrm{distilled}\ \mathrm{water} $$

$$ \mathrm{Two}\ \mathrm{g}\mathrm{rams}\ \mathrm{of}\ \mathrm{each}\ \mathrm{sample}\kern0.5em +\kern0.5em 2.5\;\mathrm{g}\ \mathrm{trypsin}\kern0.5em +\kern0.5em 1500\;\mathrm{mL}\ \mathrm{distilled}\ \mathrm{water} $$

**Fig. 1:**
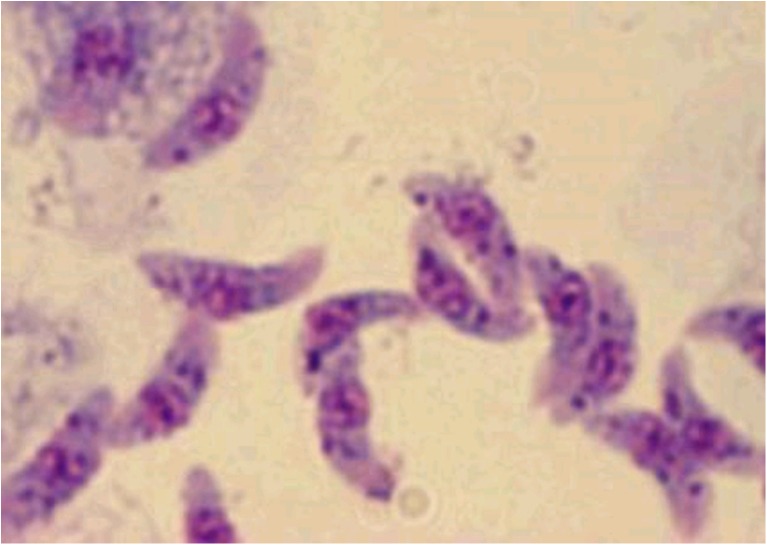
*Sarcocystis* bradyzoite by Giemsa staining method (Dob smear). (magnification ×1000)

**Fig. 2:**
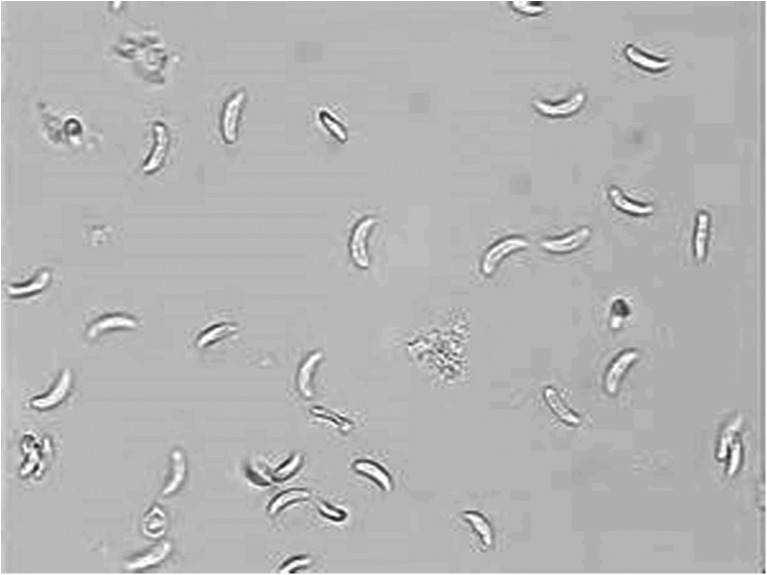
*Sarcocystis* bradyzoite by digestion method. (magnification ×400).

After digestion, all samples were incubated at 37 °C for 30 min and sieved through mesh and centrifuged at 2000 rpm for 5 min. The supernatant fluid was discarded and sediment was stained by Giemsa staining method. For *Sarcocystis* bradyzoite detection, all samples were examined by light microscope (40 and 100 magnification). *Sarcocystis* bradyzoite were shown in Figs. 1 and 2.

## Results

Our results showed that 16 (80%) of samples were infected with *Sarcocysti*s spp. (Table 1). Based on digestion method, the infection rate in hamburger, sausage, and cocktail were 87.5, 83.33, and 66.66%, respectively. The highest infestation rate was observed in hamburger of plants A and B. Whereas the lowest infection rate was observed in sausage of plant B.

**Table 1:** Infection rate of *Sarcocystis* spp. in plants A, B, and C

	Plant A		Plant B		Plant C	
Sample type	No. examined	Infected number (%)	No. examined	Infected number (%)	No. examined	Infected number (%)
Hamburger	4	4 (100)	1	1 (100)	3	2 (66.66)
Sausage	–	–	2	1 (50)	4	4 (100)
Cocktail	–	–	3	2 (66.66)	3	2 (66.66)

## Discussion

*Sarcocystis* is an obligatory intercellular parasite in mammals. Several investigations showed that there are considerable infection rate in sheep and cattle that are infected with *Sarcocystis* spp. in the entire world (Sahl Poulsen and Rune 2014; Fayer et al. 2015; Rosenthal et al. 2012; Mehdizadeh et al. 2014; Meistro et al. 2015; Hajimohammadi et al. 2014a; Jahed Khaniki and Kia 2006; Rahdar and Salehi 2011a; Prayson et al. 2008; Hosseini et al. 2007; Nematollahia et al. 2013). *Sarcocystosis* is distributed worldwide and more than 150 species of the parasite have been isolated from various domestic and wild animals. Humans are infected with this parasite as intermediate and final host in which the parasite inhabits the muscular and intestinal tract. Studies show that eating of undercooked or infected raw meat or meat products can cause infection in human. Consumption of fast foods in recent years is increasing as American people consume about five billion hamburgers annually (Gabriele et al. 2006).

Many investigations have concerned the prevalence of parasites in foods, especially *Sarcocystis* spp. In the present study, *Sarcocystis* was detected in different types of meat products. Several researches have been conducted on the prevalence of *Sarcocystis* in meat production. Jahed khaniki et al. reported that 6.25% of hamburger were positive for *Sarcocystis* cyst (Jahed Khaniki and Kia 2006). Another study has shown that 77.9% of all tested hamburger were infected with *Sarcocystis* spp. The infection rate in traditional hamburger (87%) was significantly higher than the industrial ones (67.8%) (Hajimohammadi et al. 2014a). Rahdar and Salehi demonstrated that hamburger, sausage, and hotdogs were infected with *Sarcocystis* in considerable amount in 56, 8, and 20%, respectively (Rahdar and Salehi 2011a). Prayson et al. found *Sarcocystis* spp. in two out of eight examined hamburger brands in the USA by using histological method (Prayson et al. 2008). Hosseini et al. reported occurrence of 47.9% (56 of 117) *Sarcocystis* infection, by using impression smear assay, in distributed hamburger in Tehran, Iran (Hosseini et al. 2007). Another study in Tabriz announced that the prevalence rate of *Sarcocystis* spp. in both traditional and industrial hamburger using both impression smear and peptic digestion methods was the same as 56.25% (Nematollahia et al. 2013). In the present study, we showed that digestion method is better than impression smear method in detection *Sarcocystis*. This finding agrees with other studies in Iran and the world: Bradyzoites of parasite were observed in 97.14% of animals’ digested muscles in Yazd (Hajimohammadi et al. 2014b). The current study demonstrated that peptic digestion method gave the highest rate (93.3%) followed by indirect fluorescent antibody test (IFAT) (88.6%), squeezing (81.3%), and muscle squash (81.2%) (Latif et al. 1999). Infection rate of *Sarcocystis* in cattle slaughtered in Shiraz, Iran by digestion impression methods were 100 and 99% (Shekarforoush et al. 2004).

## Conclusion

Preventing human *Sarcocystosis* will continue to rely on sanitary approaches to food preparation, but understanding the particular risks and transmission modes in the future will increasingly benefit from improved differential diagnosis based on genetic individuation. Successful methods for rendering specific diagnoses, from either tissue cysts or oocysts, are now available in places (such as China) where raw meat consumption remains prevalent among certain ethnic minorities (Rosenthal et al. 2012). People must be aware of the risk of *Sarcocystosis*. Thus, for the prevention of human infection, the meat should be frozen or cooked sufficiently before consumption.
